# Pain in Adolescence: Maternal and Paternal Factors Affecting Adolescents’ Pain in Norway—A Cross-Sectional Study

**DOI:** 10.3390/children10121915

**Published:** 2023-12-12

**Authors:** Erik Grasaas, Hilde Timenes Mikkelsen, Kristin Haraldstad, Sølvi Helseth, Milada Cvancarova Småstuen, Siv Skarstein, Gudrun Elin Rohde

**Affiliations:** 1 Department of Public Health and Nutrition, Faculty of Health and Sport Sciences, University in Agder, P.O. Box 422, 4604 Kristiansand, Norway; 2 Department of Health and Nursing Science, Faculty of Health and Sport Sciences, University in Agder, 4604 Kristiansand, Norway; hilde.e.mikkelsen@uia.no (H.T.M.); kristin.haraldstad@uia.no (K.H.); solvi@oslomet.no (S.H.); gudrun.e.rohde@uia.no (G.E.R.); 3 Department of Nursing and Health Promotion, Faculty of Health Sciences, Akershus University College of Applied Sciences, 0167 Oslo, Norway; milasm@oslomet.no (M.C.S.); siv.skartstein@oslomet.no (S.S.); 4 Department of Clinical Research, Sorlandet Hospital, 4615 Kristiansand, Norway

**Keywords:** adolescents, pain, stress, parents, health literacy

## Abstract

Background: Pain in adolescence is considered a worldwide concern. Adolescents’ pain affects family functioning. However, bidirectional associations should be considered as parental determinates such as pain, stress, and sociodemographic factors are also shown to influence pain in adolescence. Objectives: This study explored the associations between maternal and paternal sociodemographic factors, pain, and stress and adolescents’ pain, and stress on adolescents’ pain. Methods: In total, 508 school-based Norwegian adolescents with a corresponding parent were included. All adolescents completed an electronic survey during school hours, and their respective parents responded electronically. The survey included sociodemographic data, the Perceived Stress Questionnaire, and the Brief Pain Inventory. Results: Herein, 385 adolescents reported an average pain of 2.1 (SD, 1.9), and 308 of the participating parents reported an average pain of 1.6 (SD, 1.8). Regressions stratified by parental gender revealed nonsignificant associations in fathers’ study variables predicting adolescents’ pain. However, having the highest maternal educational level (*p* ≤ 0.01) and working part-time (*p* ≤ 0.01) were associated with lower pain in adolescents. Conclusions: The findings of this study demonstrated that sociodemographic factors such as high educational status in mothers and mothers working part-time were associated with lower pain in Norwegian adolescents. These findings highlight the importance of a holistic approach to pain management in adolescence.

## 1. Introduction

Pain in adolescence is common and considered a worldwide concern [1]. The commonality of pain prevalence among adolescents fluctuates in the literature, often due to differences in study groups, age variation, sample sizes, or measurements, which was identified in 1991 in the first extensive review of pain epidemiology by Goodman and McGrath [2]. In a systematic review by King et al. investigating pain in children and adolescents, 32 studies were included, and a substantial variation in the prevalence of headache, ranging from 8% to 83%, was reported [3]. Gobina and colleagues included data evidence from 42 countries and examined the prevalence of self-reported chronic pain among adolescents; they found that 44% of adolescents reported pain occurring weekly in the last 6 months [4]. In Norway, similar findings were shown wherein health surveys revealed that one-third of adolescents aged 13–18 years had pain occurring weekly for the last 3 months [5]. In a more recent study including Norwegian adolescents, 24% of adolescents reported neck/shoulder pain; these adolescents also tended to report depression and other co-occurring pain problems [6]. The high prevalence of pain in adolescence and the corresponding potential high future costs in society highlight the importance of identifying determinates for pain in adolescence.

To address pain in adolescence, the biopsychosocial model was reported to encompass a holistic understanding of pain [7]. The International Association for the Study of Pain (IASP) has implemented the biopsychosocial understanding in their definition of pain as “an unpleasant sensory and emotional experience associated with actual or potential tissue damage, or described in terms of such damage” [8]. Regarding the biological part of the model, genetic predispositions are reported to be of relevance. A systematic review revealed in twin studies that a 50% risk for developing headaches, such as tension-type headaches, migraines, or chronic, widespread pain, was related to genetic factors [9]. Moreover, it is suggested in the literature, pain is linked to a shared biological sensitivity often classified as “pain vulnerability” or “central sensitivity syndrome” [10,11,12]. 

Within families, pain may arise and continue on to the next generation, wherein both genetic and social, physiological, and cultural factors may influence the pain experience, including knowledge of relevant pain-coping strategies [13]. Lewandowski et al. investigated, in a systematic review, the family functioning of adolescents living with chronic pain. Overall findings revealed that families of adolescents with chronic pain tend to have poorer family functioning compared to healthy families [14]. However, it is important to underline the directional reciprocity of the association. It is natural to assume that adolescents’ pain affects family functioning; however, there is an increasing amount of research evidence emphasizing that parental determinates seem to highly influence pain in adolescence. Evans et al. investigated children’s psychological and physical health outcomes when living with parents with chronic pain. Their findings revealed that parental chronic pain considerably influences the lives of young children, it is yet rarely studied [15]. Further, Evans and Keenan investigated in a pilot study whether children are similarly affected when fathers and mothers experience pain. Their findings revealed that the children of mothers who experienced chronic pain reported the most psychological and physical problems, subsequent to the children of fathers who experienced chronic pain and the children participating in the control group [16]. Parental factors are reported to be associated with pain in adolescence [17,18,19]. Still, a Norwegian study revealed that chronic pain in fathers and mothers was not found to be related to child outcomes [20]. However, it was reported in a Norwegian sample that adolescents with persistent pain tend to have someone in their family with pain, indicating the importance of family history and pain management within families [21]. More research on paternal and maternal determinants of adolescents’ pain is needed. 

Several studies have shown how sociodemographic factors such as income level income and educational level (often expressed as a proxy for socioeconomical status (SES)) impact a higher risk of pain in adolescents [22,23,24]. Similar findings were reported in Norway wherein health complaints are reported more frequently among adolescents whose parents attained a lower level of education and whose perceived family economy is poor [25]. Parental knowledge of pain-coping strategies is often passed to offspring, and thus parents and especially mothers are reported to be the most important source of information for Norwegian adolescents in terms of pain management [26]. Parents of children and adolescents with pain often experience a stressful phase, and the parents’ ability to cope with the situation could be highly important [27]. According to Lazarus and Folkman, stress may be defined as “a relationship between the person and their environment that is appraised by the person as taxing or exceeding their resources and as endangering their well-being” [28].

Interestingly, it has been reported that parental factors seem to predict adolescents’ outcomes in chronic pain interventions. Murray et al. investigated which factors derive the greatest advantage from Internet-delivered interventions among adolescents with chronic pain. Their findings suggested that having parents who exhibit low levels of distress was an important predictor for lower pain in adolescents [29]. Parental distress is related to adolescents’ functioning [25]; thus, exploring different maternal and paternal factors affecting adolescents’ pain may expand our understanding. The exploration of maternal and paternal factors is needed to provide a holistic approach for both practice and policy. 

There is scarce research evidence exploring the relationships of maternal and paternal sociodemographic factors, pain, and stress with adolescents’ pain in Norway.

Thus, this paper sought to address current research gaps by using the following aim and corresponding hypothesis. 

The aim of this study was to explore the associations between maternal and paternal sociodemographic factors, pain, and stress on adolescents’ pain.

We hypothesized that maternal factors would have the strongest association with adolescents’ pain. 

## 2. Materials and Methods

### 2.1. Design

This cross-sectional study was conducted in the southeastern part of Norway as part of the “Start Young—quality of life and pain in generations” study. Start Young is a longitudinal study aimed at acquiring updated evidence about pain and HRQOL in adolescents and their respective parents/guardians [30]. This current study used baseline data collected from the end of 2018 to April 2019.

### 2.2. Study Setting

In total, 59 elementary schools covering the ninth grade from the southeastern part of Norway were invited to take part in this study. Twenty-two schools from different locations and with varied sizes agreed to participate. Due to GDPR restrictions, we were not allowed to follow up with nonresponding schools. No exclusion criteria were set. Therefore, the etiology of pain and type of pain may vary across participants. Potential participants in the present study were 1663 adolescents with their corresponding parents (mother or father) from the participating schools. At baseline, 696 adolescents (41.8%) were included, and 561 mothers or fathers responded to the questionnaire (33.7%). In total, 508 adolescent–parent dyads participated in the study (30.5%). The response rate varied from 2.9% to 71.1% across schools.

### 2.3. Study Procedures

Project members visited the participating schools about one week before data collection; at that time, the adolescents received verbal and written study information. Written information was provided to their parents. Active informed consent was obtained from both parents and adolescents. Data collection was carried out using a web-based questionnaire which the adolescents completed in the classroom during school hours. The teacher and at least one project member were present to help when needed. Parents received an e-mail with a secure link to the electronic survey and completed the survey in their spare time. A secure data server was used to store the collected data [31]. We used information from the parents’ consent forms to link the adolescents’ surveys with their parents’ surveys by generating mutual ID numbers. Study procedures were reviewed and approved by the Norwegian Centre for Research Data (NSD) with reference number 60981.

### 2.4. Measures

The adolescents’ questionnaire included questions about sociodemographic data, age, parental marital status, and other health-related questionnaires, including pain (dependent variable). 

The parental questionnaire included questions about age, work status, educational status, household income, and questions about health-related factors, including stress, and pain (independent variables).

#### 2.4.1. Pain

Pain in adolescents and in parents was measured using one question selected from the Brief Pain Inventory (BPI) which focused on average pain intensity [32]. Both parents and adolescents were asked to rate their currently subjective intensity of pain at its average [33,34]. This item is presented as a numeric rating scale from 0 to 10 where 0 represents no pain and 10 represents pain as bad as you can imagine. The Norwegian version of the BPI has been used in several studies among adolescents and adults and has shown satisfactory psychometric properties [33,35,36,37,38]. 

#### 2.4.2. Stress

Stress in parents was measured using the 30-item Perceived Stress Questionnaire (PSQ) which comprises both positively and negatively formulated items that are rated on a 4-point rating scale based upon the last four weeks [39]. Categories were recoded such that higher scores revealed higher levels of perceived stress. A PSQ total score from −30 to 90 was transformed linearly between 0 and 1 by = (raw value − 30)/90. Commonly used cutoff levels for PSQ scores between 0 and 1 are low, <0.33; medium, 0.33–0.45; moderate, 0.45–0.60; and severe, >0.60 [40]. The Norwegian version of the PSQ has shown good validity and reliability [41].

### 2.5. Statistical Analyses

All descriptive analyses were conducted using IBM SPSS Statistics for Windows, Version 25.0 (IBM Corp., Armonk, NY, USA). Descriptive measures were used to describe sociodemographic data. Continuous variables were described by presenting means and standard deviations (SDs), and categorical variables were presented with frequencies and percentages. Multivariate regression analyses were conducted between the predicting parental independent study variables (age, pain, stress, marital status, educational status, work status, and household income) and the dependent variable (adolescents’ pain). As the sociodemographic variables were included in the multivariate regression analyses, adjusting for possible confounders such as educational level was not applicable; thus, crude analyses are presented. Multivariate regression analyses were conducted using Stata Statistical Software, version 17 (StataCorp LLC, College Station, TX, USA). The first category in the independent multicategory variables was set as the reference group for the most logical comparison and interpretation of the results. For measuring the strength of the respective relationships, effect sizes were included in the multivariate regressions and expressed as Cohen’s d values with the following categories of effect: 0.2, small effect; 0.5, medium effect; and 0.8, large effect [42]. *p*-values < 0.05 were considered statistically significant and all tests were two-sided. 

## 3. Results

### 3.1. Participants

In total, 508 school-based adolescents with corresponding parents (mother or father) were included in this study, wherein 385 adolescents reported an average pain of 2.1 (SD, 1.9) and 308 of the parents reported an average pain of 1.6 (SD, 1.8). The adolescents’ ages were 13 years (1.6%), 14 years (88.2%), and 15 years (10.2%). 

### 3.2. Descriptive Data of Sociodemographic Characteristics, Pain and Stress in Parents Stratified by Gender

The mean age of the parents was 45.3 years (SD, 4.9), somewhat higher for fathers (46.6 years, SD 5.3) than mothers (44.9 years, SD 4.7). Mothers reported higher average pain (1.7, SD 1.9) than fathers (1.1, SD 1.5) and higher stress levels, 0.28 (SD 0.24) and 0.25 (SD 0.14), respectively (Table 1). The majority of parents were married or lived as cohabitants and had ≥13 years of education. Most of the fathers reported working full-time (87%) compared to 70% of the mothers. Further, 65% of the fathers reported the highest category of household income compared to 47% of the mothers. 

### 3.3. The Associations between Maternal and Paternal Sociodemographic Factors, Pain, and Stress and Adolescents’ Pain

No statistically significant associations were revealed in the reported fathers’ study variables, such as sociodemographic factors, pain, or stress, on adolescents’ pain (Table 2, all *p* > 0.05). Regarding maternal educational status, both subcategories of 13–15 years of education (B = −0.68; 95% CI [−1.26 to −0.11]) and ≥16 years of education (B = −0.81; 95% CI [1.35 to −0.27]) were statistically significantly associated with adolescents’ pain compared to ≤12 years of education and revealed effect sizes (Cohen’s d) of −0.15 and −0.21, respectively. Mothers working part-time revealed a statically significant association with adolescents ‘pain (B = −0.67; 95% CI [−1.16 to −0.17]) compared to working full-time.

## 4. Discussion

This paper sought to address current research gaps by aiming to describe maternal and paternal sociodemographic factors, pain, and stress and further explore these factors’ regarding their association with adolescents’ pain. Descriptive findings by gender revealed a homogenous parental sample with overall low pain and stress levels wherein most worked full-time and three-quarters of the study sample had over 13 years of education. Our regression analyses revealed nonsignificant associations for the fathers’ predicting study variables; however, in mothers, high educational status and working part-time were associated with lower levels of pain in Norwegian adolescents.

Our descriptive results revealed a parental group in their mid-forties with fathers on average close to a couple of years older than mothers and, given the adolescents’ age of 14 years old (range 13–15), these findings correspond closely to the average age of having children in Norway at present [43]. Although our findings demonstrated that mothers reported somewhat higher levels of perceived stress than fathers, both reported values are categorized as low [39]. From a population perspective, the vast majority fall in the lowest category for perceived stress, approximately 14% fall in the moderate category, and 3% fall in the highest category [39]. Although our study sample reported low levels of perceived stress, the age group of 35-to-54 years of age usually reports the highest scores of perceived stress compared to other age-groups [39]. 

Moreover, our findings revealed that close to 9 out of 10 fathers reported working full-time, whereas 7 out of 10 mothers reported the same. Although fewer mothers were found to be working full-time, the gap is closing in according to Statistics of Norway, which reported that the trend in Norway among mothers is an increase in full-time positions due to more flexible working conditions [44], although there might be several reasons for more mothers working part-time, such as having more responsibilities at home with children or combining part-time work with a higher educational degree. Descriptive findings of educational status revealed that more mothers than fathers have the highest educational status, which is in accordance with a recent report from the Norwegian Centre for Research on Gender Equality [45]. 

As parents’ distress has been shown to affect adolescents’ functioning [25] and even predict outcomes of adolescents’ pain-management interventions [29], our findings of a positive coefficient for perceived stress in parents with adolescents’ pain was expected as it seems logical that higher perceived stress in parents is associated with higher pain levels in adolescents. Although we did not reveal any significant associations herein, it is interesting to note that perceived stress and pain among parents revealed opposite directions of effect on adolescents’ pain, which was contrary to our expectations. Although it has been reported in Norway that paternal chronic pain is not associated with child outcomes [20], other epidemiological evidence from Norway, including data from 8200 participants, indicates that paternal chronic pain is associated with increased odds of pain in adolescents [19]. The discrepancy in the direction of the association might be explained by the fact that parents with chronic pain conditions are more likely have higher pain levels and thus wider estimates compared to our study sample of parents with overall very low pain levels.

A mother or father’s relationship with their adolescent consists of a complex interaction with several potential factors that might affect the adolescents’ pain experience, such as environmental factors, genetics, or learned pain behavior [46,47]. Hence, it is highly interesting that our regression analyses revealed all nonsignificant associations among the fathers predicting independent study variables and adolescents’ pain. These findings are in contrast with the literature, wherein higher paternal educational levels are associated with a lower degree of health complaints in adolescents [25]. Potential reasons explaining this may be due to our study sample comprising younger adolescents, higher levels of SES, and geographical differences at play, and/or our paternal study sample was too small or selected to identify differences. Research evidence reveals a higher risk of pain in adolescents coming from low SES families [22,23,24], as earlier reported in Norway [25]. Moreover, our findings of reported household income are in accordance with lower SES levels among adolescents with higher levels of pain. Although our regression did not reveal any significant association between household income and adolescents’ pain, a clear negative coefficient was revealed for all subgroups compared to the reference group (lowest household income), yielding a better outcome (lower pain in adolescence) in households with a higher income. Prospective analyses of adolescents from the North-Trøndelag Health Study (HUNT) in Norway revealed that girls and boys with a perceived low family income had 29% and 17% higher probabilities of pain, respectively, compared to adolescents with a perceived high family income [48]. However, as mothers’ and fathers’ incomes are often naturally reported together as the total household income or family economy, it is, a from a research perspective, a challenge to separately evaluate these respective variables stratified by gender and therefore fully explore their potential impacts. 

We hypothesized that maternal factors would have the strongest association with adolescents’ pain. Our regressions analyses revealed that the highest degree of education status among mothers was negatively associated with adolescents’ pain, meaning that a high maternal education status is associated with lower pain intensity levels in adolescents compared to ≤12 years of maternal education. These findings are in accordance with the literature, which reported that the lowest symptoms load of pain reported by adolescents in Norway were found for children of mothers with the highest educational status [25]. Further, the highest symptom load of pain reported by adolescents was found for children of mothers with the lowest educational status [25]. It is interesting to discuss the fact that educational status among parents seems to influence the perception of pain in adolescence regardless of the professions obtained [24,25] as neither this current study nor the literature addresses or adjusts for health-related professions. Hence, higher educational status among mothers might influence the ability to systematically search for relevant information, combined with a critical ability to consider which sources of information are relevant and thus providing the credibility needed to deliver relevant intergenerational coping strategies to their adolescents. Although many factors influence adolescents’ emotions in everyday life, such as school, friends, and social media [49,50], it seems that mothers play an important role as a source of information for Norwegian adolescents [26]. In light of our findings revealing that for mothers, working part-time mothers was associated with lower levels of adolescents’ pain, having mothers with both high educational competence and enough time seems to be important for Norwegian adolescents’ perception of pain. It is reported that maternal long hours of work are particularly harmful for children and adolescents aged 11–15 and are related to SES as less educated mothers are more often not satisfied with their hours of work, which may create difficulty in reconciling work and family life [51]. Moreover, qualitative data indicate that mothers often share their attitudes and pain management strategies with their adolescents, and that frequent interactions in terms of modeling and verbal communications are needed to provide a sort of autonomy in pain management in adolescents [52]. 

### 4.1. Strengths and Limitations

This current study comprised relatively large sample size including both adolescents and one of their corresponding parents, which should be considered a clear strength. However, more mothers (n = 393) than fathers (n = 115) participated in the study and thus could affect the statistical strengths of the gender estimates. Moreover, study variables such as household income and marital status were included in the stratified gender analyses, even though only one parent reported for the total household. However, we believed they could shed light on the participating paternal or maternal participant and serve as an important indication of SES and were therefore interesting to include. Most of the parents reported a high educational status, working full-time, and having more than NOK 750.000/year of income, which indicate a higher SES level among the study sample, and may not be representative of other populations. We do not have any additional data on mothers working part-time, which should be considered a limitation. Further, due to using the current subjective rating of average pain via the BPI, we had no insight into fluctuations in pain, type of pain, multisite pain, and the duration or frequency of previous pain experiences, which should be considered a major limitation as other covariates may have influenced the subjective rating herein. All limitations should be taken into consideration when interpreting the results of this study. Finally, it should be highlighted that this is a cross-sectional study wherein no causal interference in associations can be determined. Our direction of association is based on the rationale from research evidence together with experts within the field; however, in cross-sectional studies, there is no guarantee of the direction of association, and causality can never be statistically proven. A low participation rate in some schools, in combination with several nonparticipating schools, could imply a selection bias and thus reduces the generalizability of the findings and should be considered a limitation. The high educational level and income level revealed in this study indicate a high SES study sample, which may not be representative of the general Norwegian population or other countries and should be considered a limitation.

### 4.2. Clinical Implications

It is crucial to highlight and identify factors that may affect pain in adolescents as it provides a better understanding of how to intervene and provide a holistic pain management approach. All adolescents will experience everyday pain in varying degrees, and their pain management strategies learned from parents might be crucial in the development, maintenance, or avoidance of the perception. Hence, informing practice and policy of the importance and potential impact of parents’ determinates in adolescents who struggle with pain is important. These are important considerations to be aware of from a clinical perspective when designing and conducting pain intervention for Norwegian adolescents. 

## 5. Conclusions

This current study explored the associations between maternal and paternal sociodemographic factors, pain, and stress and adolescents’ pain and demonstrated that high educational status in mothers and mothers working part-time were associated with lower pain in Norwegian adolescents. All the fathers’ predicting study variables revealed nonsignificant associations with adolescents’ pain. Our findings indicate that higher educational competence among mothers seems to be important and beneficial for adolescents’ pain in Norway, whereas the finding that mothers working part-time has a beneficial effect on adolescents’ pain might be influenced by the high SES level of the study sample. These findings highlight the importance of understanding a holistic approach to pain management in adolescence. Future studies should investigate longitudinal and observational studies and include more fathers. 

## Figures and Tables

**Table 1:** Sociodemographic characteristics and pain and stress in parents (N = 508).

Study Variable	Parents (n = 508) (Mean/SD)	Mothers (n = 393) (Mean/SD)	Fathers (n = 115) (Mean/SD)
**Age**	45.3 (4.9)	44.9 (4.7)	46.6 (5.3)
**Pain ^a^**	1.6 (1.8)	1.7 (1.9)	1.1 (1.5)
**Stress ^b^**	0.27 (0.15)	0.28 (0.24)	0.25 (0.14)
**Marital status N (%) ^c^**			
**Married/cohabitant**	422 (83.1)	324 (82.4)	98 (85.2)
**Single/divorced**	86 (16.9)	69 (17.6)	17 (14.8)
**Educational status N (%)**			
**≤12 years of education**	117 (25.0)	100 (26.5)	27 (23.5)
**13–15 years of education**	129 (25.4)	94 (23.9)	35 (30.4)
**≥16 years of education**	252 (49.6)	199 (50.6)	53 (46.1)
**Work status N (%)**			
**Yes, full-time**	375 (73.8)	275 (70.0)	100 (87.0)
**Yes, part-time**	91 (17.8)	81 (20.6)	10 (8.7)
**No, unemployed**	42 (8.3)	37 (9.4)	5 (4.3)
**Household income N (%) ^d^**			
**≤450,000 NOK/year**	44 (8.7)	39 (9.9)	5 (4.4)
**451,000–750,000 NOK/year**	88 (17.3)	70 (17.8)	18 (15.7)
**751,000–1,000,000 NOK/year**	116 (22.8)	99 (25.2)	17 (14.8)
**>1,000,000 NOK/year**	260 (51.2)	185 (47.1)	75 (65.2)

^a^ Range 0 to 10, higher scores indicate higher pain intensity. ^b^ Range 0 to 1, higher scores indicate higher perceived stress, N = 507 (missing data for one mother). ^c^ = Dichotomized as “Married/cohabitant” or “Single/divorced” (single, divorced, or widowed) ^d^ = Recoded into four categories: “≤450,000 NOK/year” (<250,000 NOK/year, 250,000–450,000 NOK/year), “451,000–750,000 NOK/year”, “751,000–1,000,000 NOK/year”, or “>1,000,000 NOK/year.

**Table 2:** Linear regressions of parental sociodemographic factors, pain, and stress (independent variables) effects on adolescents’ pain (dependent variables), stratified by gender.

	Fathers				Mothers			
	B	95% CI	*p*-Value	Effect Size (Cohen’s d)	B	95% CI	*p*-Value	Effect Size (Cohen’s d)
Age	−0.01	−0.08 to 0.05	0.72	−0.04	−0.03	−0.07 to 0.01	0.14	−0.08
Pain	−0.21	−0.50 to 0.06	0.12	−0.19	−0.03	−0.15 to 0.08	0.55	−0.04
Stress	1.29	−1.33 to 3.91	0.10	0.09	0.89	−0.46 to 2.24	0.20	0.07
Marital status								
Married/cohabitant (ref)								
Single/divorced	0.45	−0.55 to 1.44	0.37	0.09	0.49	−0.16 to 1.15	0.14	0.10
Educational status								
≤12 years of education (ref)								
13–15 years of education	0.53	−0.47 to 1.52	0.29	0.14	−0.68	−1.26 to −0.11	0.02	−0.15
≥16 years of education	0.21	−0.76 to 1.18	0.67	0.06	−0.81	−1.35 to −0.27	<0.01	−0.21
Work status								
Yes, full time (ref)								
Yes, part time	0.55	−0.72 to 1.81	0.40	0.09	−0.67	−1.16 to −0.17	<0.01	−0.14
No, unemployed	−0.82	−2.67 to 1.01	0.38	−0.10	−0.37	−1.07 to 0.33	0.30	−0.06
Household income								
≤450,000 NOK/year (ref)								
451,000–750,000 NOK/year	0.69	−1.45 to 2.83	0.52	0.14	−0.67	−1.46 to 0.11	0.09	−0.13
751,000–1,000,000 NOK/year	0.53	−1.69 to 2.75	0.64	0.11	−0.38	−1.24 to 0.48	0.39	−0.08
>1,000,000 NOK/year	0.16	−2.05 to 2.39	0.89	0.04	−0.25	−1.13 to 0.62	0.57	−0.07

CI = confidence interval.

## Data Availability

The data presented in this study are available on request from the corresponding author. Data from this current study are not publicly available due to the General Data Protection Regulation laws but are available if permission from the Norwegian Centre for Research Data is granted.

## Abbreviations

PSQ: Perceived Stress Questionnaire; BPI: Brief Pain Inventory: SD; standard deviation: CI; confidence interval; HUNT; North-Trøndelag Health Study: SES; socioeconomic status: NSD; Norwegian Centre for Research Data.

## References

[1] Swain M.S., Henschke N., Kamper S.J., Gobina I., Ottová-Jordan V., Maher C.G. (2014). An international survey of pain in adolescents. BMC Public Health.

[2] Goodman J.E., McGrath P.J. (1991). The epidemiology of pain in children and adolescents: A review. Pain.

[3] King S., Chambers C.T., Huguet A., MacNevin R.C., McGrath P.J., Parker L., Macdonald A.J. (2011). The epidemiology of chronic pain in children and adolescents revisited: A systematic review. Pain.

[4] Gobina I., Villberg J., Välimaa R., Tynjälä J., Whitehead R., Cosma A., Brooks F., Cavallo F., Ng K., de Matos M.G. (2019). Prevalence of self-reported chronic pain among adolescents: Evidence from 42 countries and regions. Eur. J. Pain.

[5] Hoftun G.B., Romundstad P.R., Zwart J.-A., Rygg M. (2011). Chronic idiopathic pain in adolescence—High prevalence and disability: The young HUNT Study 2008. Pain.

[6] Jahre H., Grotle M., Smedbråten K., Richardsen K.R., Bakken A., Øiestad B.E. (2021). Neck and shoulder pain in adolescents seldom occur alone: Results from the Norwegian Ungdata Survey. Eur. J. Pain.

[7] Andrasik F., Flor H., Turk D.C. (2005). An expanded view of psychological aspects in head pain: The biopsychosocial model. Neurol. Sci..

[8] Merskey H. (1994). Logic, truth and language in concepts of pain. Qual. Life Res..

[9] Nielsen C.S., Knudsen G.P., Steingrímsdóttir Ó.A. (2012). Twin studies of pain. Clin. Genet..

[10] Kindler L.L., Bennett R.M., Jones K.D. (2011). Central sensitivity syndromes: Mounting pathophysiologic evidence to link fibromyalgia with other common chronic pain disorders. Pain Manag. Nurs..

[11] Burri A., Ogata S., Lachance G., Williams F. (2015). Chronic widespread pain: Clinical comorbidities and psychological correlates. Pain.

[12] Williams F.M., Spector T.D., MacGregor A.J. (2010). Pain reporting at different body sites is explained by a single underlying genetic factor. Rheumatology.

[13] Violon A. (1985). Family etiology of chronic pain. Int. J. Fam. Ther..

[14] Lewandowski A.S., Palermo T.M., Stinson J., Handley S., Chambers C.T. (2010). Systematic review of family functioning in families of children and adolescents with chronic pain. J. Pain.

[15] Evans S., Keenan T.R., Shipton E.A. (2007). Psychosocial adjustment and physical health of children living with maternal chronic pain. J. Paediatr. Child Health.

[16] Evans S., Keenan T.R. (2007). Parents with chronic pain: Are children equally affected by fathers as mothers in pain? A pilot study. J. Child Health Care.

[17] Clementi M.A., Faraji P., Poppert Cordts K., MacDougall K., Wilson A., Palermo T.M., Lewandowski Holley A. (2019). Parent Factors are Associated with Pain and Activity Limitations in Youth with Acute Musculoskeletal Pain: A Cohort Study. Clin. J. Pain.

[18] Palermo T.M., Valrie C.R., Karlson C.W. (2014). Family and parent influences on pediatric chronic pain: A developmental perspective. Am. Psychol..

[19] Hoftun G.B., Romundstad P.R., Rygg M. (2013). Association of Parental Chronic Pain with Chronic Pain in the Adolescent and Young Adult: Family Linkage Data from the HUNT Study. JAMA Pediatr..

[20] Kaasbøll J., Ranøyen I., Nilsen W., Lydersen S., Indredavik M.S. (2015). Associations between parental chronic pain and self-esteem, social competence, and family cohesion in adolescent girls and boys—Family linkage data from the HUNT study. BMC Public Health.

[21] Mikkelsen H.T., Haraldstad K., Helseth S., Skarstein S., Småstuen M.C., Rohde G. (2021). Pain and health-related quality of life in adolescents and the mediating role of self-esteem and self-efficacy: A cross-sectional study including adolescents and parents. BMC Psychol..

[22] Lachman M.E., Weaver S.L. (1998). The sense of control as a moderator of social class differences in health and well-being. J. Pers. Soc. Psychol..

[23] Kristenson M., Eriksen H., Sluiter J., Starke D., Ursin H. (2004). Psychobiological mechanisms of socioeconomic differences in health. Soc. Sci. Med..

[24] Dorner T.E., Muckenhuber J., Stronegger W.J., Ràsky E., Gustorff B., Freidl W. (2011). The impact of socio-economic status on pain and the perception of disability due to pain. Eur. J. Pain.

[25] Sæther S.M.M., Sivertsen B., Haugland S., Bøe T., Hysing M. (2018). Health complaints in late adolescence; Frequency, factor structure and the association with socio-economic status. Scand. J. Public Health.

[26] Skarstein S., Lagerløv P., Helseth S., Leegaard M. (2019). How do parents influence their adolescents’ use of over-the-counter analgesics: A review of the current literature. J. Clin. Nurs..

[27] Palermo T.M., Eccleston C. (2009). Parents of children and adolescents with chronic pain. Pain.

[28] Lazarus R.S., Folkman S. (1986). Cognitive theories of stress and the issue of circularity. Dynamics of Stress: Physiological, Psychological and Social Perspectives.

[29] Murray C.B., de la Vega R., Loren D.M., Palermo T.M. (2020). Moderators of Internet-Delivered Cognitive-Behavioral Therapy for Adolescents with Chronic Pain: Who Benefits from Treatment at Long-Term Follow-Up?. J. Pain.

[30] Mikkelsen H.T., Haraldstad K., Helseth S., Skarstein S., Småstuen M.C., Rohde G. (2020). Health-related quality of life is strongly associated with self-efficacy, self-esteem, loneliness, and stress in 14–15-year-old adolescents: A cross-sectional study. Health Qual. Life Outcomes.

[31] University of Oslo Services for Sensitive Data (TSD). https://www.uio.no/english/services/it/research/sensitive-data/index.html.

[32] Cleeland C.S., Ryan K.M. (1994). Pain assessment: Global use of the Brief Pain Inventory. Ann. Acad. Med. Singap..

[33] Klepstad P., Loge J.H., Borchgrevink P.C., Mendoza T.R., Cleeland C.S., Kaasa S. (2002). The Norwegian brief pain inventory questionnaire: Translation and validation in cancer pain patients. J. Pain Symptom Manag..

[34] Cleeland C.S., Ryan K. (1991). The brief pain inventory. Pain Res. Group.

[35] Winger A., Kvarstein G., Wyller V.B., Sulheim D., Fagermoen E., Småstuen M.C., Helseth S. (2014). Pain and pressure pain thresholds in adolescents with chronic fatigue syndrome and healthy controls: A cross-sectional study. BMJ Open.

[36] Larsen S.M., Ramstad K., Terjesen T. (2021). Hip pain in adolescents with cerebral palsy: A population-based longitudinal study. Dev. Med. Child Neurol..

[37] Garnaes K.K., Mørkved S., Salvesen Ø., Tønne T., Furan L., Grønhaug G., Vasseljen O., Johannessen H.H. (2021). What factors are associated with health-related quality of life among patients with chronic musculoskeletal pain? A cross-sectional study in primary health care. BMC Musculoskeletal. Disorders.

[38] Jelsness-Jørgensen L.-P., Moum B., Grimstad T., Jahnsen J., Opheim R., Berset I.P., Hovde Ø., Torp R., Frigstad S.O., Huppertz-Hauss G. (2016). Validity, Reliability, and Responsiveness of the Brief Pain Inventory in Inflammatory Bowel Disease. Can. J. Gastroenterol. Hepatol..

[39] Levenstein S., Prantera C., Varvo V., Scribano M., Berto E., Luzi C., Andreoli A. (1993). Development of the Perceived Stress Questionnaire: A new tool for psychosomatic research. J. Psychosom. Res..

[40] Kocalevent R.-D., Levenstein S., Fliege H., Schmid G., Hinz A., Brähler E., Klapp B.F. (2007). Contribution to the construct validity of the Perceived Stress Questionnaire from a population-based survey. J. Psychosom. Res..

[41] Østerås B., Sigmundsson H., Haga M. (2018). Psychometric Properties of the Perceived Stress Questionnaire (PSQ) in 15–16 Years Old Norwegian Adolescents. Front. Psychol..

[42] Cohen J. (1988). Statistical Power Analysis for the Behavioral Sciences.

[43] Statistics of Norway Rekordlav Fruktbarhet for Tredje år på Rad. https://www.ssb.no/befolkning/artikler-og-publikasjoner/rekordlav-fruktbarhet-for-tredje-ar-pa-rad.

[44] Statistics of Norway Færre Barn, Flere i Fulltidsjobb. Arbeid og Lønn. https://www.ssb.no/arbeid-og-lonn/artikler-og-publikasjoner/faerre-barn-flere-i-heltidsjobb.

[45] Reisel L., Seehuus S. (2022). Flere Kvinner Enn Menn i Høyere Utdanning.

[46] Higgins K.S., Birnie K.A., Chambers C.T., Wilson A.C., Caes L., Clark A.J., Lynch M., Stinson J., Campbell-Yeo M. (2015). Offspring of parents with chronic pain: A systematic review and meta-analysis of pain, health, psychological, and family outcomes. Pain.

[47] Stone A.L., Wilson A.C. (2016). Transmission of risk from parents with chronic pain to offspring: An integrative conceptual model. Pain.

[48] Jahre H., Grotle M., Småstuen M., Guddal M.H., Smedbråten K., Richardsen K.R., Stensland S., Storheim K., Øiestad B.E. (2021). Risk factors and risk profiles for neck pain in young adults: Prospective analyses from adolescence to young adulthood—The North-Trøndelag Health Study. PLoS ONE.

[49] Beyens I., Pouwels J.L., van Driel I.I., Keijsers L., Valkenburg P.M. (2020). The effect of social media on well-being differs from adolescent to adolescent. Sci. Rep..

[50] Lennarz H.K., Hollenstein T., Lichtwarck-Aschoff A., Kuntsche E., Granic I. (2019). Emotion regulation in action: Use, selection, and success of emotion regulation in adolescents’ daily lives. Int. J. Behav. Dev..

[51] Mendolia S. (2011). Maternal Working Hours and the Well-Being of Adolescent Children. SSRN Electron. J..

[52] Hatchette J.E., McGrath P.J., Murray M., Finley G.A. (2006). Maternal influences in adolescents’ pain self-management: A qualitative investigation. Vulnerable Child. Youth Stud..

